# Effects of glucocorticoids on interstitial glucose concentrations in individuals with hematologic cancer and without known diagnosis of diabetes: a pilot study

**DOI:** 10.31744/einstein_journal/2022AO8031

**Published:** 2022-07-04

**Authors:** Marcos Tadashi Kakitani Toyoshima, Priscilla Cukier, Alexandre Barbosa Câmara de Souza, Juliana Pereira, Ana Oliveira Hoff, Marcia Nery

**Affiliations:** 1 Hospital das Clínicas Faculdade de Medicina Universidade de São Paulo São Paulo SP Brazil Instituto do Câncer do Estado de São Paulo Octavio Frias de Oliveira, Hospital das Clínicas, Faculdade de Medicina, Universidade de São Paulo, São Paulo, SP, Brazil.; 2 Hospital das Clínicas Faculdade de Medicina Universidade de São Paulo São Paulo SP Brazil Hospital das Clínicas, Faculdade de Medicina, Universidade de São Paulo, São Paulo, SP, Brazil.; 3 Hospital das Clínicas Faculdade de Medicina Universidade de São Paulo São Paulo SP Brazil Laboratórios de Investigação Médica, Hospital das Clínicas, Faculdade de Medicina, Universidade de São Paulo, São Paulo, SP, Brazil.

**Keywords:** Blood glucose, Glucose, Hematologic neoplasms, Lymphoma, Hyperglycemia, Glucocorticoids, Diabetes mellitus, Drug therapy

## Abstract

**Objective:**

To analyze interstitial glucose behavior during glucocorticoid use in non-diabetic patients receiving chemotherapy for hematologic malignancies.

**Methods:**

Prospective pilot study carried out to assess interstitial glucose levels in 15 non-diabetic individuals with hematologic malignancies who received glucocorticoids in combination with chemotherapy. The FreeStyle Libre flash monitoring system (Abbott Diabetes Care) was used for up to 14 days to measure interstitial glucose.

**Results:**

Median age and body mass index were 53 (42-61) years and 25 (23-28) kg/m^2^ respectively. Interstitial glucose levels >180mg/dL lasting at least one hour were detected in 60% of participants. Interstitial glucose profile parameters (median and peak interstitial glucose levels and percentage of time during which interstitial glucose levels were >180mg/dL) were significantly (p<0.01) higher during glucocorticoid use (115mg/dL, 218mg/dL and 10% respectively) than after glucocorticoid discontinuation (97mg/dL, 137mg/dL and 0% respectively). Mean interstitial glucose levels increased in the afternoon and at night during glucocorticoid use.

**Conclusion:**

This pilot study was the first to evaluate interstitial glucose levels in non-diabetic individuals using glucocorticoids in treatment of hematologic cancer. Glucocorticoid use during chemotherapy significantly increases interstitial glucose levels in these patients.

## INTRODUCTION

The coexistence of cancer and *diabetes mellitus* has been reported for more than 50 years and the relationship between both diseases has been increasingly studied.^(1)^ There is evidence that hyperglycemia or hyperinsulinemia may facilitate cancer cell growth. Epidemiological studies suggest a relation between diabetes and the incidence and prognosis of some types of cancers, including liver, pancreas, endometrial, colorectal, breast and bladder cancer.^(1,2)^ Epidemiological studies have shown that diabetes increases cancer patient mortality.^(1)^ Even in non-diabetic cancer patients, hyperglycemia significantly increases the risk of death.^(2)^ However, the impact of hyperglycemia on response to cancer treatment and survival has not been well established so far.^(1,2)^ According to the consensus of the American Diabetes Association and the American Cancer Society,^(1)^ associations between diabetes and cancer should be investigated according to the site of primary cancer rather than as a whole, as different types of cancers have different biological and clinical behavior.

The management of diabetes in cancer patients is complex, since several factors, such as glycemic target, nutritional status, antidiabetic therapy, use of glucocorticoids, chemotherapy and immunotherapy, must be accounted for.^(3)^ Glucocorticoids are often used in clinical oncology as an adjunct to chemotherapy due to their antiemetic, immunosuppressive and anti-inflammatory effects.^(4)^ However, these drugs have several common metabolic side effects, including hypertension, osteoporosis and diabetes. Glucocorticoids may aggravate hyperglycemia in diabetic patients, precipitate symptoms in prediabetic patients or cause glucocorticoids-induced diabetes.^(5-7)^ Glucocorticoids are the main cause of drug-induced hyperglycemia in cancer patients and may lead to worse quality of life due to symptoms such as fatigue, polyuria, dehydration and increased risk of infection.^(3,4)^

The behavior of hyperglycemia in oncologic patients treated with glucocorticoids requires better understanding.^(8)^ Almost all studies addressing glucocorticoids-induced hyperglycemia during chemotherapy in cancer patients used fasting or random point-of-care capillary blood glucose (BG) measurements to detect episodes of hyperglycemia. However, these studies did not cover the entire period of exposure to glucocorticoids.^(9)^ An alternative to capillary BG would be to use continuous interstitial glucose (IG) monitoring devices.^(10)^

Continuous glucose monitoring systems (CGMS) consist of a sensor inserted into the subcutaneous tissue, which measures IG concentration at regular intervals for a set period of time, according to each device. The first CGMSs were approved for use around the year 2000. Such systems have been increasingly used to support outpatient management of diabetes ever since. Continuous glucose monitoring systems enable a better understanding of BG behavior throughout the day and prevent hypo or hyperglycemia.^(10,11)^

The paucity of data describing the behavior of hyperglycemia in cancer patients on glucocorticoids and the availability of CGMS devices have motivated the investigation of the role of this device in management of these patients.

A limited number of donated CGMS devices were used in this study. In order to avoid the inclusion of a wide range of oncological diseases, hematologic neoplasms treated with glucocorticoids in combination with chemotherapy were selected.

## OBJECTIVE

To analyze interstitial glucose behavior during glucocorticoid use in patients with hematologic malignancies receiving chemotherapy.

## METHODS

### Study design

A prospective pilot study was carried out to assess interstitial glucose levels in individuals with hematologic malignancies receiving glucocorticoids combination with chemotherapy. Chemotherapy regimens and glucocorticoids (dose, duration and drug) were prescribed by the Hematology team. Medical records were analyzed for collection of demographic data, medical history of diabetes, fasting BG values prior to chemotherapy, specific hematologic cancer diagnosis, type and dose of glucocorticoid and chemotherapy regimen. The study was named Libre Onco Study and carried out in compliance with the Declaration of Helsinki. Procedures involving human beings were approved by the Research Ethics Committee of *Faculdade de Medicina da Universidade de São Paulo* (# 2.180.632, CAAE: 71548617.5.0000.0065), which was responsible for research projects of *Instituto do Câncer do Estado de São Paulo Octavio Frias de Oliveira - Hospital das Clínicas da Faculdade de Medicina da Universidade de São Paulo.*

### Device use

The FreeStyle Libre flash monitoring system (Abbott Diabetes Care, Witney, United Kingdom) was used for up to 14 days to measure IG concentrations. Studies with this device had already validated its use in clinical practice.^(12,13)^ A total of 20 donated devices were received. Interstitial glucose measurements were analyzed after sensor removal.

### Outcome measures

The primary outcome was the difference between median IG concentration during and after glucocorticoid use. Other IG profile parameters, such as peak IG concentration and the percentage of time during which IG was higher than 180mg/dL (>10mmol/L) during IG monitoring, were evaluated at the same time points. Interstitial glucose behavior during and after glucocorticoid use was compared between patients using prednisone or dexamethasone, including the circadian rhythm of IG. Laboratory and demographic variables commonly associated with hyperglycemia were analyzed.

### Study population

Non-diabetic individuals aged over 18 years with hematologic neoplasms who were receiving chemotherapy and glucocorticoids were included. Pregnant women and individuals who were unable to understand how to use the CGMS device were not eligible for participation in the study. Individuals receiving exclusively palliative care or who were not on glucocorticoids while using the device were excluded from the analysis. Individuals were screened at the hematology outpatient clinic. Recruitment was carried out until enrollment reached 20 eligible individuals. Devices were then placed. Participants signed an informed consent form prior to enrollment.

### Statistical analysis

Continuous variables with normal distribution were expressed as means and standard deviations (SD). Continuous variables with non-normal distribution were expressed as medians, 25^th^ and 75^th^ percentiles. Categorical variables were expressed as counts and percentages.

Graphs with daily median IG concentrations were constructed to display IG behavior during and after glucocorticoid use. Differences detected throughout the day were assessed using one-way ANOVA.

In order to assess IG patterns after glucocorticoid use, days were divided into two 12-hour periods (a.m. and p.m.) or four 6-hour periods (dawn: 12:00 a.m. - 05:59 a.m.; morning: 6:00 a.m. - 11:59 a.m.; afternoon: 12:00 p.m. - 05:59 p.m. and night: 06:00 p.m. -11:59 p.m.). Curves were built for graphical representation of IG concentrations per hour and for analysis of circadian patterns of IG concentration.

Sub-analyses were performed according to type of glucocorticoid (dexamethasone or prednisone). Bonferroni correction was used for multiple comparisons.

The χ^2^ test or the Fisher’s exact test was used to compare nominal variables between groups during and after glucocorticoids use. The Student *t*-test and the Mann-Whitney U test were used to compare continuous variables with and without normal distribution respectively.

Pearson correlation coefficients were used to investigate correlations between median IG concentration, peak IG concentration, the percentage of time during which IG was >180mg/dL (>10mmol/L) over the course of follow up and factors potentially related to changes in IG concentration. Dexamethasone doses were converted into equivalent prednisone doses (0.75mg of dexamethasone = 5mg of prednisone).

The p values <0.05 were considered significant. Statistical analyses were performed using Stata Statistical Software, version 15 (College Station, TX, USA).

## RESULTS

### Clinical characteristics of participants

A total of 20 study participants were selected to use the IG monitoring device and signed the informed consent form. Five individuals were excluded from analysis (two due to device detachment, two who were not taking glucocorticoids while using the device and one due to discontinuation of chemotherapy and initiation of exclusive palliative care).

Baseline characteristics of the remaining 15 participants, oncological parameters and duration of IG monitoring are shown in table 1. Median age was 53 (42-61) years, 60% of participants were males and median body mass index (BMI) was 25 (23-28) kg/m^2^. Fasting BG measurements prior to chemotherapy were obtained in 80% of individuals; median BG concentration was 100 (93-111) mg/dL. Hematologic malignancies were as follows: 12 cases of non-Hodgkin’s lymphomas (NHL), one case of Hodgkin’s lymphoma, one case of Castleman’s disease and one case of Waldenström’s macroglobulinemia. Chemotherapy regimens are shown in table 1.

**Table 1:** Clinical characteristics of participants, oncological parameters and duration of interstitial glucose monitoring

Variables	Study participants
Age (years)	53 (42-61)
Sex, n (%)	
Female	6 (40)
BMI (kg/m^2^)	25 (23-28)
Fasting glycemia (mg/dL)	94 (84-106)
Neoplasias, n (%)	
NHL	12 (80)
Hodgkin’s lymphoma	1 (7)
Castleman’s disease	1 (7)
Waldenström’s macroglobulinemia	1 (7)
Chemotherapy regimen, n (%)	
R-CHOP	6 (40)
Modified IVAC	2 (13.3)
CVP	1 (6.7)
R-CVP	2 (13.3)
R-Hyper-CVAD	1 (6.7)
GDP	1 (6.7)
GIV	1 (6.7)
GEMOX	1 (6.7)
Glucocorticoid type, n (%)	
Prednisone	9 (60)
Dexamethasone	6 (40)
Duration of glucocorticoid use (days)	5 (5-7)
Total glucocorticoid dose* (mg)	533 (533-667)
Duration of IG monitoring during glucocorticoid use (days)	5 (5-6)
Duration of IG monitoring without glucocorticoid use (days)	4 (2-7)

* equivalent dose of prednisone. Data expressed as n (%) or median (percentiles 25^th^ and 75^th^).

BMI: body mass index; NHL: non-Hodgkin’s lymphoma; Chemotherapy regimens: R-CHOP: rituximab, cyclophosphamide, doxorubicin, vincristine, dexamethasone and prednisone; Modified IVAC: etoposide ifosfamide/Mesna and cytarabine; CVP: cyclophosphamide, vincristine, dexamethasone, and prednisone; R-CVP: rituximab, cyclophosphamide, vincristine, dexamethasone, and prednisone; R-Hyper CVAD: rituximab, cyclophosphamide, dexamethasone, doxorubicin, vincristine, methotrexate and cytarabine; GDP: gemcitabine, dexamethasone and cisplatin; GIV: gemcitabine, ifosfamide/Mesna and vinorelbine; GEMOX: gemcitabine and oxaliplatin.

### Interstitial glucose

In 9 (60%) out of 15 participants IG levels were higher than 180mg/dL (>10mmol/L) for at least one hour. The median time during which IG levels remained higher than 180mg/dL (>10mmol/L) was 3 (0-5) hours, and the median peak IG concentration was 218 (171-259) mg/dL. Individuals remained hyperglycemic ten percent of the time while using glucocorticoids. Interstitial glucose profile parameters (median IG concentration, peak IG concentration and percentage of time during which IG was >180mg/dL) were significantly (p<0.01) higher during glucocorticoids use [115mg/dL (102-142), 218mg/dL (171-259) and 10% (0-16) respectively] than after glucocorticoids use [97mg/dL (88-105), 137mg/dL (124-171) and 0% (0-1) respectively] (Table 2).

**Table 2:** Interstitial glucose concentration during and after glucocorticoid use

Variables	All the time	During GC use	After GC use	p value
IG (mg/dL)				
During follow-up	105 (94-111)	115 (102-142)	97 (88-105)	<0.01
a.m.	98 (91-115)	101 (96-117)	92 (90-99)	0.05
p.m.	122 (100-146)	141 (122-169)	104 (97-114)	<0.01
12:00 a.m.-05:59 a.m.	94 (85-108)	101 (83-114)	93 (86-101)	0.42
06:00 a.m.-11:59 a.m.	98 (93-108)	101 (97-120)	95 (88-102)	0.10
12:00 p.m.-5:59 p.m.	122 (104-159)	141 (123-172)	105 (97-113)	<0.01
6:00 p.m.-11:59 p.m.	119 (103-166)	150 (128-167)	103 (97-117)	<0.01
Peak IG (mg/dL)	171 (138-218)	218 (171-259)	137 (124-171)	<0.01
Percentage of time with IG ≥180mg/dL (%)	3 (0-5)	10 (0-16)	0 (0-1)	<0.01

Data expressed as n (%) or median (percentiles 25^th^ and 75^th^).

GC: glucocorticoid; IG: interstitial glucose.

**Figure 1:**
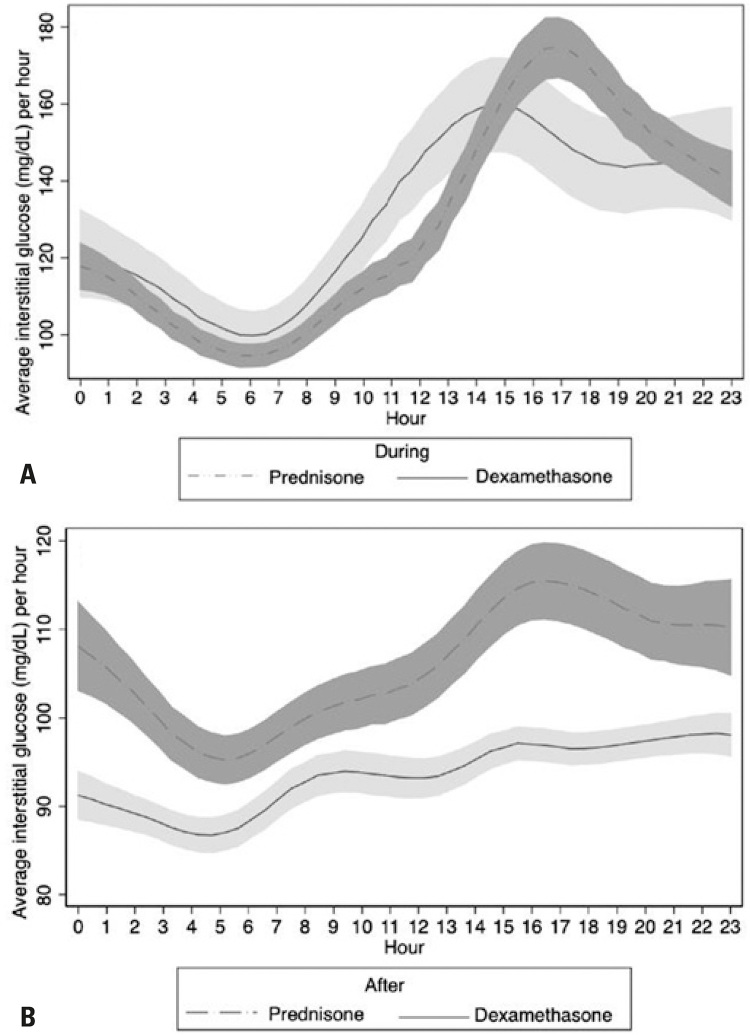
Pooled interstitial glucose concentrations per hour of the day (circadian pattern of glucose concentration) during (A) and 24 hours after (B) dexamethasone or prednisone use

Glucocorticoid doses used during chemotherapy did not differ significantly between the prednisone and the dexamethasone groups. Still, the circadian pattern of IG concentration increased in the afternoon and at night. Mean IG concentrations were higher in participants using prednisone relative to those using dexamethasone at 5:00p.m. (182.2±62.9mg/dL and 144.5±68.6mg/dL respectively; p=0.04) and 6:00p.m. (172.5±53.2mg/dL and 138.4±60.2mg/dL respectively; p=0.03) (Figure 1A).

The duration of glucocorticoid use was longer in the prednisone relative to the dexamethasone group (6.65±2.10 and 4.39±2.7 days respectively; p<0.01). Interstitial glucose concentrations decreased in both groups after glucocorticoids were discontinued. However, IG concentrations were higher in individuals using prednisone relative to those using dexamethasone. During the first 24 hours after glucocorticoid discontinuation, mean IG concentrations were 93.5±16mg/dL and 105.3±22.7mg/dL (Dexamethasone and Prednisone Group respectively; p<0.01) (Figure 1B). Other parameters after glucocorticoid discontinuation were also worse in individuals using prednisone (Table 3).

**Table 3:** Comparison of interstitial glucose concentrations during and after prednisone or dexamethasone use

Variables	During use			After use		
	Dexa	Pred	p value	Dexa	Pred	p value
Peak IG (mg/dL)	210.5 (162-225)	218 (200-259)	0.479	131 (84-138)	157 (130-188.5)	0.093
Percentage of time with IG ≥180 mg/dL, (%)	6 (0-26)	11 (2-15)	0.858	0 (0-0)	0 (0-0.5)	0.204
IG at follow-up (mg/dL)	122 (94-142)	110 (108-133)	0.814	87.5 (69-97)	101.5 (95.5-112.5)	0.027
IG: a.m.	108 (98-130)	101 (96-116)	0.443	89 (88-93)	93 (92-108)	0.121
IG: p.m.	137.5 (100-175)	141 (125-166)	0.906	97 (97-107)	107 (100-118)	0.415
IG: 12:00 a.m.-5:59 a.m.	104 (90-115)	100 (82-105)	0.317	83.5 (71-86)	95 (93-111)	0.013
IG: 6:00 a.m.-11:59 a.m.	108 (97-130)	98 (97-117)	0.442	91.5 (69-95)	102 (92-105.5)	0.070
IG: 12:00 p.m.-5:59 p.m.	138 (110-183)	144 (123-171)	0.906	97 (96-106)	109 (100-114)	0.255
IG: 6:00 p.m.-11:59 p.m.	136 (111-167)	154 (136-166)	0.479	100 (96-103)	116 (98-118)	0.515
Total dose of GC (mg, equivalent dose of prednisone)	667 (400-666)	533 (533-533)	0.463			

Data expressed as median (percentiles 25^th^ and 75^th^). Dexa: dexamethasone. GC: glucocorticoid; IG: interstitial glucose; Pred: prednisone.

**Figure 2:**
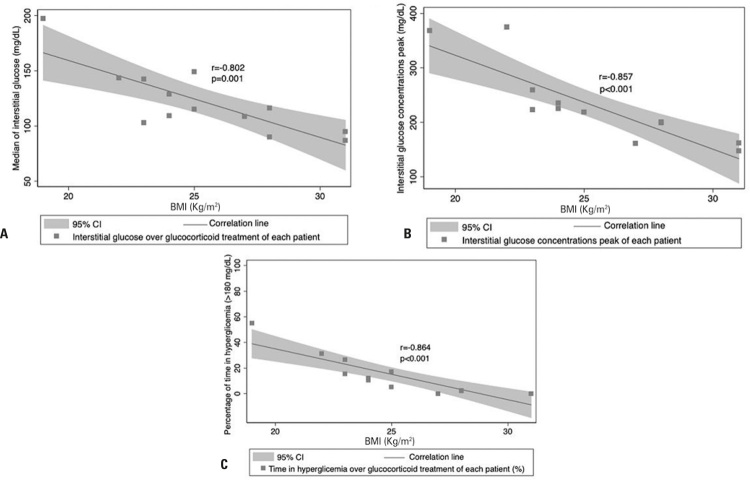
Body mass index and interstitial glucose parameters in non-diabetic individuals with hematological neoplasms undergoing chemotherapy combined with glucocorticoids. Correlations between body mass index and median interstitial glucose (A), peak interstitial glucose concentration (B) and percentage of time during which interstitial glucose concentration was at least 180mg/dL (C) 95% confidence interval

Sex, age, fasting BG prior to chemotherapy and total glucocorticoid dose were not significantly correlated with IG profile parameters. Body mass index was strongly and inversely correlated with median IG concentration (Figure 2A), peak IG concentration (Figure 2B) and percentage of time during which IG levels were >180mg/dL (>10mmol/L) (Figure 2C) (r=-0.802, p=0.001; r=-0.857, p<0.001 and r=-0.864, p<0.001 respectively).

95%CI: 95% confidence interval; BMI: body mass index.

## DISCUSSION

This study was the first to examine IG levels in non-diabetic individuals using glucocorticoids in hematologic cancer treatment. Studies assessing IG in individuals using glucocorticoids are still scarce. One study investigated 16 non-diabetic individuals with gynecologic cancer who received dexamethasone combined with chemotherapy consisting of carboplatin and paclitaxel and were using the Medtronic iPRO2™ CGMS device. Almost all individuals developed hyperglycemia, defined as IG levels higher than 200mg/dL (11.1mmol/L) in the first day of treatment.^(9)^ In another study, which did not involve oncology, diabetic and non-diabetic individuals with chronic obstructive pulmonary disease (COPD) treated with prednisolone were investigated. In that study, 53% of non-diabetic individuals who received high doses of glucocorticoids had a hyperglycemic episode (glucose concentration of at least 200mg/dL or 11.1mmol/L).^(14)^

Glucocorticoids are important drugs in cancer treatment. However, they are associated with increased insulin resistance in all insulin-sensitive tissues and interfere with insulin secretion by pancreatic beta cells.^(15,16)^

In this study, 60% of the non-diabetic individuals with hematological malignancies who were using glucocorticoids had IG concentrations higher than 180mg/dL (>10mmol/L) for at least one hour and remained hyperglycemic 10% of the time while using glucocorticoids. These findings suggest the prevalence of glucocorticoids-induced hyperglycemia is high.

Most studies on glucocorticoids-induced hyperglycemia have used fasting or postprandial BG to detect episodes of hyperglycemia. There is great heterogeneity between existing studies in several aspects, such as dose and duration of glucocorticoids use, definition of glucocorticoid-induced diabetes and severity of individuals. In these studies, the incidence of glucocorticoids-induced diabetes ranged from 2% to 65%.^(6,17-20)^ Studies investigating glucocorticoid-induced hyperglycemia during chemotherapy in hematological cancer patients are scarce. An observational study with 278 patients with acute lymphocytic leukemia (ALL) treated with hyper-CVAD (cyclophosphamide, dexamethasone, doxorubicin, vincristine, methotrexate and cytarabine) revealed that 37% of patients had BG ≥200mg/dL (11.1mmol/L) in at least two measurements. In that study, patients who developed hyperglycemia also had less time to complete remission, increased mortality and increased risk of infections.^(20)^ A study showed that hyperglycemia (BG >180mg/dL or 10mmol/L) was related to shorter overall survival and progression-free survival in patients with ALL.^(21)^

Most individuals in this study had NHL and 40% of them received rituximab, cyclophosphamide, doxorubicin, vincristine, dexamethasone and prednisone (R-CHOP) chemotherapy. In fact, R-CHOP is the most commonly used chemotherapy regimen to treat aggressive forms of NHL. Reviewing the literature, an observational study with individuals with NHL who were treated with R-CHOP or R-EPOCH (R-CHOP plus etoposide) chemotherapy revealed that almost 50% of them had at least one episode of random hyperglycemia (BG ≥200mg/dL or 11.1mmol/L).^(6)^

The use of BG measurements to screen for glucocorticoids-induced hyperglycemia does not cover the entire period of exposure to glucocorticoids.^(9)^ One advantage of using an IG monitoring device over BG is precisely the ability to monitor glucose levels throughout the day. This analysis also showed that glucocorticoids raises IG concentrations in the afternoon and early evening. Similar findings have been reported in a study with individuals who used glucocorticoids due to COPD.^(14)^ That study also supported the predominant effect of glucocorticoids on postprandial IG in diabetic and non-diabetic individuals. This study did not include meal time data. Therefore, meal times could not be correlated with IG levels.

With the advent of CGMSs, new metrics of appropriate glycemic control have emerged, such as glycemic variability.^(22)^ Glycemic variability is a measure of the magnitude of glucose fluctuations throughout the day or over a certain period of time. The impact of glycemic variability on clinical outcomes is still poorly studied.^(19)^ In an experimental *in vitro* study, wider glycemic variability was associated with increased local invasion and metastasis in pancreatic adenocarcinoma.^(23)^

In clinical practice, many individuals treated with glucocorticoids during chemotherapy do not receive medical advice regarding glycemic control measures. In outpatient settings, fasting serum glucose is usually requested and measurements made in the morning. However, hyperglycemia usually occurs late in the afternoon and in the evening, since glucocorticoids are often administered in the morning, especially prednisone. Therefore, hyperglycemic episodes may not be detected. Inpatients should be screened for glucocorticoid-induced hyperglycemia using point-of-care BG, monitored for at least 48 hours and treated whenever hyperglycemia is confirmed.^(24)^

Data in this study revealed normalization of IG concentrations following glucocorticoid discontinuation. However, these individuals must be carefully monitored due to the increased risk of diabetes development.^(25)^

In spite of the small number of patients, risk factors for glucocorticoid-induced hyperglycemia were evaluated and correlations between BMI and IG concentration detected. Higher BMI is associated with increased risk of glucocorticoid-induced diabetes.^(5)^ Although our findings revealed a strong and inverse correlation between BMI and IG parameters, there were no obese individuals in this study. Lower BMI may reflect cancer cachexia, which is associated with lower performance status, greater intolerance to cancer treatment and increased mortality. Individuals with cancer cachexia have higher endogenous glucose production, increased gluconeogenesis and greater insulin resistance. A chronic inflammatory state that results in increased insulin resistance and pancreatic beta cell dysfunction has also been demonstrated.^(26,27)^ Normal fasting BG can be used to distinguish between glucocorticoid-induced hyperglycemia secondary to cancer cachexia and type 2 diabetes phenotype. Fasting BG concentrations tend to be normal in patients with cachexia and higher in patients with type 2 diabetes.^(26,27)^

The choice of glucocorticoid in chemotherapy regimens is generally arbitrary and scientific evidence of better results that might support preference is lacking.^(28)^ Dexamethasone is considered a long-acting glucocorticoid, whereas prednisone is an intermediate-acting glucocorticoid.^(29)^ Our findings also revealed a slower decline IG concentrations after glucocorticoid discontinuation in patients using prednisone than in those using dexamethasone.

In a prospective study with children with ALL who were randomized to use of prednisone or dexamethasone, the incidence of severe hyperglycemia was higher in children using dexamethasone relative to prednisone.^(30)^ A meta-analysis comparing the use of dexamethasone and prednisone for induction therapy in ALL failed to detect differences regarding glucocorticoid-induced diabetes.^(31)^

The benefit of appropriate glycemic control in patients with diabetes or stress hyperglycemia is well known. In inpatient settings, the diagnosis and treatment of acute hyperglycemia is of paramount importance to reduce morbidity, length of hospital stay, intensive care unit admission and in-hospital mortality. Failure to recognize and treat this condition may increase the risk of hospital infections by up to 5.8 times,^(32)^ with negative impacts on the progression of patients with acute myocardial infarction,^(33)^ poorer functional recovery after stroke^(34)^ and increased risk of thrombotic events,^(35)^ among other negative consequences, particularly in non-diabetic patients. In non-diabetic patients with acute hyperglycemia, in-hospital mortality rates are almost ten times higher than in normoglycemic patients.^(36)^ Studies addressing hyperglycemia prevention in patients who will be prescribed high doses glucocorticoids and the consequences of treating hyperglycemia are scarce.^(37)^ Prospective studies are needed for deeper understanding of the clinical implications and benefits of preventing or treating glucocorticoid-related hyperglycemia.^(7,37)^

This study has limitations: it is a pilot cross-sectional study and laboratory tests, such as glycated hemoglobin, were not performed prior to chemotherapy. Attempts were made to attenuate this deficiency by comparing periods with and without glucocorticoid use. In this pilot study, sample size was relatively small and may not have been enough to show correlations. This study did not assess oncological outcomes, such as therapeutic response or tumor remission. Further studies are needed to determine the clinical significance of findings presented. However, this study emphasizes the fact that fasting blood glucose measurements may not detect glycemic changes associated with the use of glucocorticoids. Use of interstitial glucose monitoring systems or simple blood glucose measurements before dinner may help detect individuals at risk of developing hyperglycemia.

## CONCLUSION

In conclusion, this pilot study was the first to examine interstitial glucose concentrations in non-diabetic individuals who used glucocorticoids in treatment of hematological cancer. In these patients, glucocorticoid use significantly increases interstitial glucose. Studies assessing interstitial glucose concentrations are very important for a better understanding of the effects of glucocorticoids in diabetic and non-diabetic cancer patients and may support treatment optimization and improve oncological outcomes. Devices that measure interstitial glucose are useful tools for blood glucose monitoring during use of high doses of glucocorticoids. Large prospective studies addressing interstitial glucose behavior in diabetic and non-diabetic individuals using high doses of glucocorticoids are warranted. The impacts of treating glucocorticoid-induced hyperglycemia also remain to be determined.
